# The Impact of COVID-19 Lockdown on Service Utilization Among Chronic Disease Patients in South Africa

**DOI:** 10.1177/11786329231215040

**Published:** 2023-11-29

**Authors:** Micheal Kofi Boachie, Mariana Khoza, Susan Goldstein, Maggie Munsamy, Karen Hofman, Evelyn Thsehla

**Affiliations:** 1 Discipline of Public Health Medicine, School of Nursing and Public Health, Howard College, University of KwaZulu-Natal, Durban, South Africa; 2 SAMRC/Wits Centre for Health Economics and Decision Science—PRICELESS SA, School of Public Health, Faculty of Health Sciences, University of the Witwatersrand, Johannesburg, South Africa; 3 National Department of Health, Pretoria, South Africa

**Keywords:** CCMDD, COVID-19, chronic diseases, interrupted time series, South Africa

## Abstract

**Introduction:**

Globally, the COVID-19 pandemic has brought many disruptions in health service delivery. Evidence show that the pandemic has negatively affected routine healthcare utilization such as maternal and child health services, but the literature on the effect on non-communicable diseases (NCDs) is scant in South Africa. These disruptions can have long-term health and economic implications for patients.

**Objective:**

To estimate the impact of COVID-19 lockdown on service utilization among chronic disease patients in South Africa using administrative data.

**Methods:**

Using monthly data from the Centralized Chronic Medication Dispensing and Distribution (CCMDD) program database covering November 2018 to October 2021, we examined the effects of COVID-19 lockdown on utilization among patients receiving antiretroviral therapy (ART) medication only (ART-only), patients receiving both ART and NCD medication (ART + NCD), and patients receiving NCD medications only (NCD-only). We employed segmented interrupted time series approach to examine the changes. We stratified the analysis by socioeconomic status.

**Results:**

We found that, overall, the lockdown was associated with increased utilization of CCMDD services by 10.8% (95% CI: 3.3%-19%) for ART-only and 10.3% (95% CI: 3.3%-17.7%) for NCD-only patients. The increase in utilization was not different across socioeconomic groups. For patients receiving ART + NCD medications, utilization declined by 56.6% (95% CI: 47.6%-64.1%), and higher reductions occurred in low SES districts.

**Conclusion:**

Patients should be educated about the need to continue with utilization of disease programs during a pandemic and beyond. More efforts are needed to improve service use among patients with multi-morbidities.

## Background

In late 2019, COVID-19 emerged as one of the leading public health threats that would soon cause significant morbidity and mortality across the globe.^1,2^ As of 03 March 2022, 5.97 million lives have been lost to COVID-19 worldwide with about 100 000 of these deaths occurring in South Africa.^3^ The highly infectious nature of the disease and the lack of adequate health infrastructure including intensive care units (ICUs) caused many countries to implement lockdown restrictions. The aim of these restrictions was to halt the spread of the disease and to allow governments to equip their health facilities to deal with the emerging situation. Large gatherings, travel, work, and school attendance, for instance, were restricted in over 100 countries, causing many people to be housebound.^4^

In South Africa, a phased lockdown with 5 levels that prohibited all non-essential economic activities was implemented from 27 March 2020.^4^ This included restrictions on public transport and sales of alcohol and tobacco. Likewise, schools and all non-essential business activities including movement were also restricted. At the strictest lockdown (level 5), individuals were required to remain in their place of residence, with the exception of ‘performing an essential service, obtaining an essential good or service, collecting a social grant, pension, or seeking emergency, life-saving, or chronic medical attention.^5^ Access to and delivery of healthcare services were allowed given its essential nature.^4^

The public health benefits of lockdown measures have been documented.^6-9^ In some African countries, however, no clear pattern exist for definite conclusions to be made about the impact or effectiveness of lockdown interventions.^10^ The restrictions have been shown to have had huge social and economic implications,^10^ especially in low- and middle-income countries, where between 20% and 55% of GDP is produced in the informal sector.^11^

In addition, morbidity and mortality from acute conditions requiring immediate or ongoing care has been shown together with reductions in maternal and child health services due to social fear and reduced access to public health services for patients with other conditions.^5,12-17^ Reduced access due to re-assignment of staff to help in the COVID-19 response and the fear among the general population of contracting COVID-19 may also have affected service utilization for chronic patients. In the WHO Africa region, only 12% of countries reported not re-assigning NCD staff to help in the COVID-19 response.^18^ Indeed, experience shows that resources are diverted when interventions for specific diseases are introduced, as was the case in South Africa during the measles campaign. The introduction of the campaign was associated with significant disruption in routine care, with declines in immunization coverage, antenatal visits, and contraceptive use.^19^

While some studies in South Africa have shown that no change occurred in total healthcare utilization in some rural areas, child healthcare visits reduced due to lockdown in other settings.^5,14^ For HIV services, existing patients continued to receive ART services. However, the lockdown affected testing and ART initiations.^20^ The long-term implications could impact population health from less severe conditions arising due to reduced primary and secondary prevention. Furthermore, patients with chronic conditions such as diabetes and hypertension whose treatment may not be priorities during lockdowns may experience reduction in healthcare use, which may cause severe complications leading to death. Aside from worsening of existing conditions, such patients may develop new diseases or even die from the coronavirus.^21^

One way to improve service delivery for chronic disease patients is through medication management systems. The United Kingdom’s (UK) National Health Service (NHS) introduced a medication collection system (known as the Chronic Medication Service) to allow patients to collect medications for long-term conditions such as diabetes, chronic obstructive pulmonary disease (COPD), and hypertension.^22^ A similar system, Centralized Chronic Medication Dispensing and Distribution (CCMDD) program, exist in South Africa to improve medication access to chronic disease patients.^23^ While these programs aim to improve patients’ access to medicines, lockdown restrictions may affect usage of such services.

This study seeks to explore the impact of COVID-19 induced lockdown measures on the use of chronic disease services in South Africa. Thus, our main research questions are (1) What is the impact of COVID-19 associated lockdown on healthcare utilization among chronic disease patients? (2) Did the reduction in utilization, if any, differ by socioeconomic status? To answer the first question, we hypothesized that COVID-19 induced lockdown was associated with reduced utilization among chronic diseases patients under the CCMDD program. We also hypothesized that reduction in service utilization differed by according to socio-economic status. The analyses are important to inform policy about chronic disease service delivery in South Africa.

## Methods

### Data description

To assess the impact of lockdown measures on healthcare use, the study uses administrative data from the CCMDD program. Thus, this study is a retrospective analysis of administrative data on the CCMDD program. The CCMDD is an alternate access to chronic medication program that was implemented in 2014. The aim of the program is to improve access to medicines and enhance patient experiences. CCMDD services are available to all diagnosed patients receiving care in the public sector who meet a defined criteria such as 2 consecutive fasting plasma glucose levels and 2 consecutive blood pressure readings showing normal for NCD patients.^23^ In 2019, the program covered about 2.07 million people^23-26^ suffering from diseases such as HIV, hypertension and diabetes. Through the program, patients collect dispensed chronic medications (e.g., ART (antiretroviral therapy), anti-hypertensives, diabetes treatment, and lipid-lowering medication)) in their communities (closest to them), instead of having treatment dispensed at clinics which may be far from their residence. Patients who have been diagnosed, are stable and referred by healthcare professionals in the public health sector receive their medication from the community Pick-up-Point (PuP). Contracted service providers manage the collection of the scripts, dispensing, and delivers it to community pickup points.^23-25^ The community pickups include private pharmacies, doctor’s rooms, containers, smart lockers (“Smart Locker” PuPs), and community-based organizations. In 2019, there were 3436 health facilities and 2037 PuPs under the program.^23,26^ Patients return to the clinic for a review and re-referral (if necessary) into CCMDD every 6 months. If unwell, patients can return to clinics at any time for medical attention before the sixth month.^26^ At the same time, clinical review was reduced to once per year.

Aggregate weekly CCMDD dataset (September 2018-October 2021) covering utilization in 46 districts across 8 provinces was obtained, in a Microsoft Excel format, from the South African National Department of Health. The provinces with data included Gauteng, Eastern Cape, Free State, KwaZulu-Natal, Limpopo, North-West, Northern Cape, and Mpumalanga. The data included registered/enrolled patients and active patients, with no information on patient characteristics such as sex and age (see Liu et al^23^ for details on CCMDD data). The other variables in the dataset were provinces, districts, and week during which the data were captured onto the system. There were 1.5 million active patients in the CCMDD program in January 2018.^23^ Due to missing data in the first few months, we analyzed data between November 2018 and October 2021, with each district having 36 monthly observations each for ART-only, ART + NCD, and NCD-only. District level population estimates were obtained from Government of South Africa municipalities’ website.^27^ There is no information regarding the patient population that are suffering from HIV/AIDS and NCDs in every district. We assume that the total population of the district provides the eligible patients.

### Patient categories in the CCMDD dataset

#### Registered/enrolled patients

This is the number of enrolled patients since the start of the CCMDD program. It includes active patients (ie, patients currently receiving medication), dormant patients (ie, patients whose prescriptions have lapsed and patients who have not collected Patient Medicine parcel (PMP) at a pickup point for 2 consecutive cycles), closed-out patients (ie, patients deactivated or deregistered from CCMDD program and patients with dormant profiles (>6 months) as well as patients registered in 2014/2015 but never collected a PMP at a pickup point) and deactivated patients (ie, patients deactivated from CCMDD program at all public health establishments). Thus, the enrolled or the registered patients may consist of demised patients, patients actively collecting medication, and patients not actively collecting medication.

### Active patients

These are patients with a current valid prescription for 6 months and includes active patients in waiting. It is a subset of enrolled or registered patients described above. Components in this group are active patients receiving medications for HIV only (ART-only), HIV with other chronic conditions (ART + NCD), and patients suffering from chronic non-communicable conditions (e.g., diabetes and hypertension) only (NCD-only). That is, ART-only refers to active patients receiving ART medications only; ART + NCD, active patients receiving both ART and chronic medications; NCD-only, active patients receiving chronic medications only. We note that the analysis does not sum ART-only and NCD-only variables to produce ART + NCD. It is possible that different people simultaneously receiving ART and NCD medication.

Due to the absence of data on the number of pickups or visits and the pre-packed medicines delivered for each person, we use the number of patients (headcount) with active prescription to make inferences about utilization and pickups under the CCMDD. Thus, trends in the number of active patients on the register is used to study the trends in pickups or visits.

### Data analysis

We aggregated the weekly data by month at the district level for ART-only, ART + NCD, and NCD-only. The active patients at any particular date includes existing patients brought forward on that date plus any new registrations/referrals on that date.

To assess the effect of phased lockdown (April-September 2020) on active registrations, a panel interrupted time series (ITS) segmented regression is used. Segmented ITS approach is a quasi-experimental research design with a potentially high degree of internal validity in cases where multiple observations on the variable of interest exist for pre- and post-intervention periods.^28-30^ The approach or its variants has been used recently to study the impact of COVID-19 restrictions on healthcare utilization in China,^31^ the UK,^16^ and South Africa^5,20^ and alcohol purchases in the UK.^32^ The estimated regression equation follows an approach used in China for similar purpose^31^:

(1) $$\begin{array}{c} ln(E(Y_{it}))=\beta_{0i}+\beta_{1i}T_{it}+\beta_{2}X_{it}+\beta_{3}X_{it}T_{it}\\ +\beta_{m}M+offset(\ln\left(Pop_{it}\right))+\varepsilon_{it}\ldots \end{array}$$

where $Y_{it}$ is the count outcome variable (number of active chronic patients on the CCMDD register) measured at each equally spaced time point *t* for each location (ie, district) *i*. $Y_{it}$ is thus the cumulative number of active patients. $T_{it}$ is the time since the start of the study (November 20) capturing the structural trend or growth rate in service utilization, independently from the lockdown. $X_{it}$ is an indicator variable representing the lockdown (lockdown takes 1 for April-September 2020, otherwise 0). $X_{it}T_{it}$ is an interaction term, capturing the change in trend or growth in outcome for the post lockdown period and $\varepsilon_{it}$ is an error term. $Pop_{it}$ is the population in the catchment area (ie, district). Note that the sum of B_1_ and B_3_ yields the post-intervention slope.

We run separate regressions for each “wealth” or socioeconomic (SES) group to assess change in utilization in these groupings. The SES is calculated from the South African Multiple Deprivation Index scores produced at the district level in 2011.^33^ The Multiple Deprivation Index is a measure designed to track how deprived communities are. The index scores are constructed by weighting 4 main domains namely income, employment, education, and living environment. Detailed methodology on the construction of the multiple deprivation index scores are provided by the South African Social Policy Research Institute (SASPRI).^33^ The larger the score the more deprived a community is. These scores were then re-categorized into wealth quintile using Stata *xtile* routine command. Afterwards, the wealth quintiles were coded by assigning the values of 1 to 5 so that it reflects the SASPRI’s classification. We then linked the wealth quintile to the CCMDD dataset.

To establish the impact of the lockdown on service utilization, we used mixed-effects negative binomial segmented ITS regression technique. STATA routine command me*nbreg* was used to obtain the results. Monthly dummies (M) were used to account for potential seasonality in service utilization. A random intercept and random slope on the lockdown variable ( $X_{it}$ ) were included. This approach has been used to assess the impact of COVID-19 associated restrictions on healthcare usage in China.^31^

### Sensitivity analysis: Relaxing the assumption of linearity

The ITS analysis assumes different linear pre- and post-slopes. However, the trends might be non-linear in nature for the outcome of interest.^34^ Following a previous study,^34^ we estimated a second model by modifying equation 1 where time was included as a covariate to capture linear trend and a restricted cubic spline transformation of the time variable was used to allow for a non-linear trend using 4 knots. The coefficients of the splines were then tested using the Chi-square test under the null hypothesis of existence of linear relationship between time and ART-only, ART + NCD and NCD-only utilization. A non-linearity of the time effect can be explained by the existing or occurring effect of other time-varying factors, like changes in the number of patients preferring public health services and even the number of patients meeting the criteria for CCMDD registration. Because of the strict eligibility criteria for CCMDD registration, not all chronic disease patients are registered which may make trends non-linear.

## Results

Overall, there were 1656 observations for 46 districts in 8 provinces (36 monthly observations for each of the 46 districts) between November 2018 and October 2021. KwaZulu-Natal and Eastern Cape accounted for 24% and 17% of the total number of observations respectively, while the province with the lowest share was Mpumalanga (6.52%). Based on SASPRI’s multiple deprivation index, 40% of the districts were in the bottom category of the socioeconomic status, whereas 41% were in the top category (Table 1).

**Table 1.** Sample characteristics.

Province	Number of observations (%)	Districts names	Socioeconomic status**
Eastern Cape	288 (17.39)	A Nzo DM	Poorest
		Amathole DM	Poorest
		Buffalo City MM	Rich
		C Hani DM	Rich
		Joe Gqabi DM	Rich
		N Mandela Bay MM	Richest
		O.R. Tambo DM	Poor
		Sarah Baartman DM	Middle-income
Free state	180 (10.87)	Fezile Dabi	Richest
		Lejweleputswa	Poorest
		Mangaung	Poor
		T Mofutsanyana	Rich
		Xhariep	Richest
Gauteng	180 (10.87)	Ekurhuleni	Richest
		Johannesburg	Rich
		Sedibeng	Richest
		Tshwane	Middle-income
		West Rand	Rich
KwaZulu-Natal	396 (23.91)	Amajuba DM	Middle-income
		Harry Gwala DM	Richest
		King Cetshwayo DM	Poor
		UMgungundlovu DM	Middle-income
		Ugu DM	Poor
		Umkhanyakude DM	Poorest
		Umzinyathi DM	Poorest
		Uthukela DM	Poor
		Zululand DM	Poorest
		eThekwini MM	Richest
		iLembe DM	Poor
Limpopo	180 (10.87)	Capricorn DM	Middle-income
		Mopani DM	Rich
		Sekhukhune DM	Poorest
		Vhembe DM	Poor
		Waterberg DM	Middle-income
Mpumalanga	108 (6.52)	Ehlanzeni DM	Richest
		G Sibande DM	Middle-income
		Nkangala DM	Richest
North-West	144 (8.7)	Bojanala Platinum DM	Middle-income
		Dr K Kaunda DM	Poorest
		Ngaka Modiri Molema DM	Rich
		Ruth Segomotsi Mompati DM	Poorest
Northern Cape	180 (10.87)	Frances Baard	Poor
		J T Gaetsewe	Richest
		Namakwa	Poor
		Pixley ka Seme	Rich
		ZF Mgcawu	Middle-income

** SES constructed based on Noble et al.^34^

### Summary statistics of the variables (headcount)

**Figure 1.**
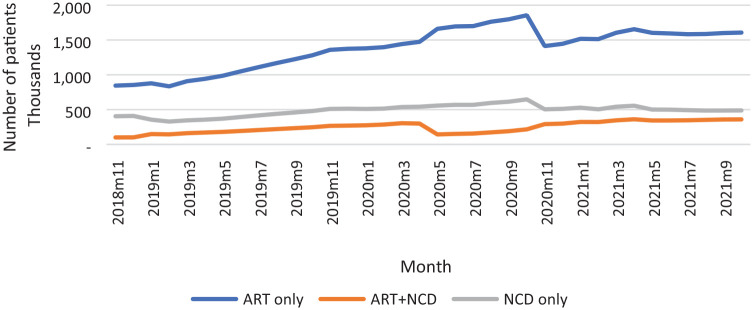
Number of patients with active prescription on the register per month, 2018-11 to 2021-10.

In November 2018, about 2.17 million patients had enrolled in the CCMDD program of which 62% were active. At the end of October 2021, 4.73 million patients had registered on the program since its inception, while 52% (about 2.45 million) of these patients were actively receiving ART-only, ART + NCD, and NCD-only medications. The composition of active patients in November 2018 were 63% for ART-only and 30% for NCD-only. By December 2019, the number of active patients had reached 2.2 million with 24% and 13%, respectively, receiving medication for NCD-only and ART + NCD. The trends in the actual number of patients with active prescriptions on the register are summarized in Figure 1.

At the national level, the average monthly utilization rate for ART-only increased from 202 per 10 000 population during pre-lockdown (ie, Nov 2018-Mar 2020) to 278 per 10 000 population during the lockdown (April-Sep 2020). The trends were similar for ART + NCD and NCD-only (see Table 2).

**Table 2.** Mean utilization rate (per 10 000 population) before and during the lockdown.

ART-only	Nov 2018-Mar 2020				April 2020-Sep 2020			
	Mean	SE	[95% Conf.	Interval]	Mean	SE	[95% Conf.	Interval]
National	202	5.63	190.76	212.88	278.82	12.12	254.95	302.68
Eastern Cape	92	4.19	83.97	100.54	121.91	8.67	104.48	139.35
Free state	155	10.73	133.50	176.17	202.17	20.24	160.78	243.57
Gauteng	140	5.22	129.33	150.09	198.48	7.57	183.00	213.96
KwaZulu-Natal	446	5.76	434.44	457.18	591.27	8.50	574.30	608.25
Limpopo	148	5.36	137.72	159.02	222.28	10.75	200.28	244.27
Mpumalanga	255	10.03	235.06	275.36	405.57	21.41	360.40	450.74
North-West	87	4.16	78.70	95.32	118.21	7.61	102.45	133.96
Northern Cape	63	4.48	53.77	71.57	108.42	12.06	83.76	133.09
ART + NCD	Nov 2018-Mar 2020				April 2020-Sep 2020			
	Mean	SE	[95% Conf.	Interval]	Mean	SE	[95% Conf.	Interval]
National	39.17	1.13	36.95	41.38	30.24	1.66	26.98	33.50
Eastern Cape	19.34	0.93	17.50	21.19	29.05	1.83	25.37	32.73
Free state	29.35	1.86	25.65	33.04	46.73	3.73	39.09	54.36
Gauteng	31.56	1.84	27.90	35.22	44.02	2.93	38.02	50.02
KwaZulu-Natal	77.62	2.59	72.51	82.73	32.89	4.64	23.62	42.17
Limpopo	15.03	0.74	13.55	16.50	7.24	1.54	4.08	10.40
Mpumalanga	45.55	2.10	41.32	49.78	18.00	5.24	6.96	29.05
North-West	38.70	2.34	34.04	43.37	54.52	4.07	46.11	62.94
Northern Cape	24.39	1.85	20.70	28.08	6.95	2.45	1.93	11.97
NCD-only	Nov 2018-Mar 2020				April 2020-Sep 2020			
	Mean	SE	[95% Conf.	Interval]	Mean	SE	[95% Conf.	Interval]
National	86.91	2.19	82.62	91.21	108.54	4.07	100.54	116.55
Eastern Cape	60.33	2.20	55.97	64.69	70.13	3.77	62.53	77.72
Free state	61.34	4.13	53.12	69.56	86.02	7.19	71.31	100.72
Gauteng	38.46	4.44	29.64	47.29	48.90	5.33	37.99	59.80
KwaZulu-Natal	160.44	4.26	152.04	168.84	191.15	8.29	174.59	207.71
Limpopo	53.29	2.71	47.91	58.67	80.51	4.21	71.90	89.12
Mpumalanga	44.94	2.04	40.84	49.03	68.98	3.24	62.14	75.81
North-West	77.15	2.55	72.06	82.24	83.98	3.21	77.34	90.62
Northern Cape	108.31	5.99	96.41	120.22	141.87	10.22	120.96	162.78

### COVID-19 lockdown and CCMDD service utilization

Table 3 reports the regression results (incidence rate ratios (IRR)) from the negative binomial segmented ITS analysis, showing the impact of the lockdown on CCMDD service utilization.

**Table 3.** Regression results on the impact of COVID-19 lockdown on CCMDD utilization.

	ART-only	ART + NCD	NCD-only
	IRR	IRR	IRR
Time-trend	1.033*** [95% CI: 1.030,1.037] (0.002)	1.057*** [95% CI: 1.051,1.063] (0.003)	1.028*** [95% CI: 1.023,1.033] (0.002)
Intervention^+^	1.108*** [95% CI: 1.033,1.189] (0.040)	0.434*** [95% CI: 0.359,0.524] (0.042)	1.103*** [95% CI: 1.033,1.177] (0.037)
Pre-post-slope difference	0.963*** [95% CI: 0.959,0.969] (0.003)	0.938*** [95% CI: 0.929,0.947] (0.004)	0.969*** [95% CI: 0.962,0.977] (0.004)
Constant	0.01*** [95% CI: 0.006,0.015] (0.002)	0.002*** [95% CI: 0.001,0.003] (0.0003)	0.005*** [95% CI: 0.004,0.007] (0.001)
Observations	1656	1656	1656
Month dummies	Yes	Yes	Yes

Abbreviation: IRR: incident rate ratios.

Standard errors in parentheses.

ART only refers to active patients receiving antiretroviral therapies (ART) medications only.

ART + NCD, active patients receiving both ART and chronic medications.

NCD only, active patients receiving chronic medications only.

*** *P* < .01.

+ April-September 2020 lockdown.

At the baseline (before lockdown), monthly active ART-only, ART + NCD, and NCD-only utilization rates (per 10 000 population) were estimated to be 99.32 (95% CI: 55.18-143.46), 17.49 (95% CI: 10.87-24.11), and 49.85 (95% CI: 35.27-64.43), respectively. These baseline utilization rates were increasing significantly (*P* < .001) by 3.3%, 5.7%, and 2.8% monthly for ART-only, ART + NCD, and NCD-only, respectively. The significant IRR for time trend and post-slope difference showed a significant month-to-month change in CCMDD service utilization before and after the April to September 2020 lockdown (Table 3).

The immediate impact of the lockdown restrictions on CCMDD utilization was positive for 2 outcomes variables. The results showed that ART-only monthly utilization rate increased by 10.8% (95% CI: 3.3% to 19%). For NCD only, the increase was 10.3% (95% CI: 3.3% to 17.7%). The average number of active patients receiving ART + NCD medications monthly which stood at approximately 18 patients per 10 000 population before the lockdown decreased by about 57%. These changes were statistically significant at conventional levels. The change in trend between pre and post lockdown was: ART only (−3.7%), NCD only (−3.1%), and ART + NCD (−6.2%).

In Table 4, the impact of lockdown segregated by socioeconomic status of the districts is presented. For monthly ART-only, baseline visits averaged 94 per 10 000 population for all income groups. The baseline utilization rate trended upwards by an average of 4% per month in all quintiles (Table 4). Although the immidiate effect of the lockdown was positive for all quintiles, it was statistically insignificant by wealth or income disaggregation.

**Table 4.** Impact of lockdown restrictions by socioeconomic status of districts.

	ART-only					ART + NCD					NCD-only				
	Quintile														
	1	2	3	4	5	1	2	3	4	5	1	2	3	4	5
Time-trend	1.028*** (0.003)	1.031*** (0.003)	1.030*** (0.003)	1.035*** (0.004)	1.043*** (0.004)	1.056*** (0.006)	1.052*** (0.006)	1.049*** (0.007)	1.068*** (0.007)	1.064*** (0.007)	1.020*** (0.004)	1.031*** (0.003)	1.019*** (0.003)	1.026*** (0.006)	1.045*** (0.006)
95% CI	1.021 -1.034	1.025 -1.038	1.023 -1.036	1.028 -1.042	1.036 -1.051	1.044 -1.069	1.040 -1.064	1.036 -1.062	1.055 -1.082	1.050 -1.077	1.013 -1.028	1.025 -1.038	1.013 -1.026	1.015 -1.037	1.033 -1.058
Intervention^+^	1.060 (0.071)	1.167 (0.111)	1.163 (0.176)	1.101 (0.081)	1.067 (0.163)	0.495*** (0.104)	0.317*** (0.057)	0.339*** (0.077)	0.608*** (0.116)	0.449*** (0.094)	1.056 (0.073)	1.124 (0.082)	1.126* (0.077)	1.091 (0.115)	1.118 (0.096)
95% CI	0.930 -1.209	0.969 -1.405	0.865 -1.565	0.954 -1.272	0.791 -1.439	0.328 -0.746	0.222 -0.452	0.217 -0.529	0.418 -0.885	0.298 -0.675	0.923 -1.209	0.974 -1.297	0.985 -1.288	0.887 -1.341	0.945 -1.323
Pre-post-slope difference	0.976*** (0.005)	0.949*** (0.005)	0.958*** (0.005)	0.979*** (0.006)	0.956*** (0.006)	0.946*** (0.009)	0.930*** (0.009)	0.928*** (0.010)	0.947*** (0.010)	0.937*** (0.010)	0.979*** (0.006)	0.949*** (0.005)	0.961*** (0.005)	0.993 (0.009)	0.961*** (0.010)
95% CI	0.966 -0.986	0.939 -0.959	0.948 -0.968	0.968 -0.990	0.945 -0.967	0.928 -0.964	0.912 -0.948	0.909 -0.947	0.927 -0.966	0.917 -0.956	0.967 -0.990	0.939 -0.960	0.950 -0.971	0.975 -1.012	0.942 -0.980
Constant	0.012*** (0.003)	0.010*** (0.004)	0.009*** (0.004)	0.008*** (0.002)	0.008*** (0.003)	0.002*** (0.001)	0.002*** (0.001)	0.002*** (0.001)	0.001*** (0.000)	0.002*** (0.000)	0.006*** (0.001)	0.006*** (0.001)	0.007*** (0.001)	0.004*** (0.002)	0.003*** (0.001)
95% CI	0.007 -0.021	0.004 -0.023	0.004 -0.024	0.005 -0.013	0.003 -0.018	0.001 -0.003	0.001 -0.003	0.001 -0.004	0.001 -0.002	0.001 -0.003	0.005 -0.008	0.005 -0.009	0.005 -0.010	0.002 -0.009	0.002 -0.006
Observations	324	324	324	324	360	324	324	324	324	360	324	324	324	324	360

+ April to September 2020; Standard errors in parentheses. *P < .1. ***P < .01. Numbers 1 to 5 represent quintile 1 to quintile 5.

For ART + NCD, utilization averaged 20 visits (per 10 000 population) monthly for all quintiles (except the fourth quintile with a rate of 10 visits) at the baseline. The pre-lockdown growth in trend averaged about 5% for all quintiles. Service utilization by patients taking ART + NCD medication declined by between 39% and 68%, with the highest reduction occurring in districts in the second and third quintiles during the lockdown (Table 4).

In the case of NCD-only medications, there were more visits per 10 000 population in the first and second quintiles (60 visits) and third quintile (70 visits), at the baseline. The richest quintile had the lowest utilization (30 visits/10 000 population). While the introduction of the lockdown showed positive effect on utilization for all quintiles, the effect was statistically insignificant.

For all outcomes, the chi-square test on the cubic splines rejects the existence of linear trend. Thus, service use under CCMDD appears to be non-linear and to the effects of unaccounted factors in the linear model which could modify the trend in utilization, such as patient and program characteristics. The immediate effect of the lockdown (Table 5) is given as: ART (IRR = 1.091, *P* < .001), ART + NCD (IRR = 0.537, *P* < .001), and NCD-only (IRR = 1.011, *P* > .1). The finding on increased utilization for ART-only reduces to 9.1%, while that on NCD-only reduces to 1.1% but statistically insignificant once the linearity assumption is relaxed. For ART + NCD, the lockdown was associated with 46% decline in service use. Though at different IRR, the positive association between lockdown and the CCMDD usage appears to be the same in both linear and non-linear models.

**Table 5.** COVID-19 lockdown and CCMDD utilization, allowing for non-linear trends.

	ART-only	ART + NCD	NCD-only
	IRR	IRR	IRR
Time-trend	1.033 *** [95% CI: 1.025, 1.041] (0.004)	1.069*** [95% CI: 1.054, 1.085] (0.008)	1.012*** [95% CI: 1.002, 1.023] (0.005)
Intervention^+^	1.091** [95% CI: 1.018, 1.169] (0.039)	0.537*** [95% CI: 0.456, 0.632] (0.045)	1.011 [95% CI: 0.949, 1.076] (0.032)
Pre-post-slope difference	0.978* [95% CI: 0.954, 1.003] (0.012)	0.894*** [95% CI: 0.854, 0.936] (0.021)	0.953** [95% CI: 0.922, 0.987] (0.017)
Constant	0.010*** (95% CI: 0.006, 0.016] (0.002)	0.002*** [95% CI: 0.001, 0.002] (0.0003)	0.005*** [95% CI: 0.004, 0.007] (0.001)

* *P* < .1. **P < .05. ***P < .01.

## Discussion

**Figure 2.**
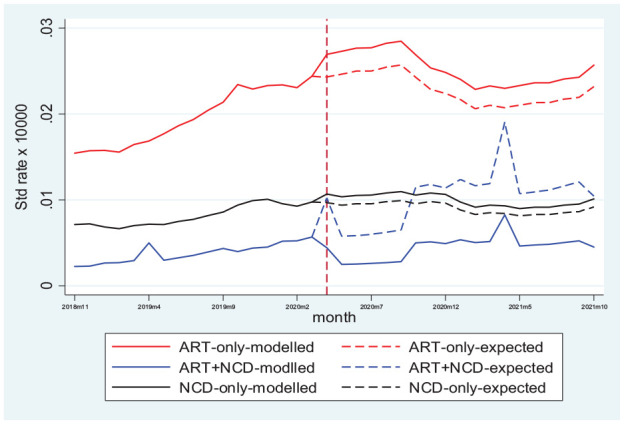
Predicted and expected mean number of patients with active prescription per 10 000 population), 2018-11 to 2021-10. Vertical dash line depicts start of lockdown.

In 2020, the emergence of COVID-19 resulted in lockdowns across the world, including South Africa. Our analyses of the CCMDD data show a significant positive association between the lockdown and service utilization for ART-only and NCD-only medication, while utilization decreased for ART + NCD medications. Although the lockdown was associated with increased utilization for ART-only and NCD-only, pre-lockdown monthly growth in utilization had still not been restored at the end of October 2021 (Figure 2). Similarly, post-lockdown trend for ART + NCD medications was −0.83% monthly compared to 5.7% before the lockdown was implemented. Disaggregation of the data by SES showed no significant variation in CCMDD service utilization across quintiles (except for ART + NCD).

The increase in utilization may be due to the use of community PuPs such as smart lockers, which health professionals saw as an avenue to reduce non-emergency visits in the hospitals. They referred more patients to PuPs in an attempt to prepare for the pandemic. Prior to the lockdown, over half of ART patients preferred collecting their medications from their local community PuPs than clinics due to convenience and shorter waiting time,^35^ and these PuPs did not require any staff contact during collection.^36^ As part of the pandemic preparedness, about 18 000 “Smart Locker” PuPs were installed in all participating provinces to ensure minimal interpersonal contact during the COVID-19 lockdown restrictions.^36^ Patients already using in-facility PuPs were shifted to these smart lockers, while clinical visits were reduced from twice to once a year. The number of patients using these lockers increased from 75 000 in April 2020 to 102 000 in September 2020 for all provinces.^36^ In KwaZulu-Natal Province, a qualitative evaluation has found that patients valued the convenience and the ease brought by the smart lockers.^37^ Our results on ART service usage are consistent with a previous study in South Africa highlighting continued collection of medications among HIV patients in 2020 even in the presence of lockdown.^20^ In the United States of America (Madison, Wisconsin), adherence to asthma and chronic obstructive pulmonary disease (COPD) medication increased by about 15% during the pandemic,^38^ whereas a significant reduction occurred in primary care services for asthma exacerbations in England.^16^ While medications requiring face-to-face consultations declined, that of type 2 diabetes, hypertension, and mental health conditions recorded no decline in prescriptions.^17^

**Figure 3.**
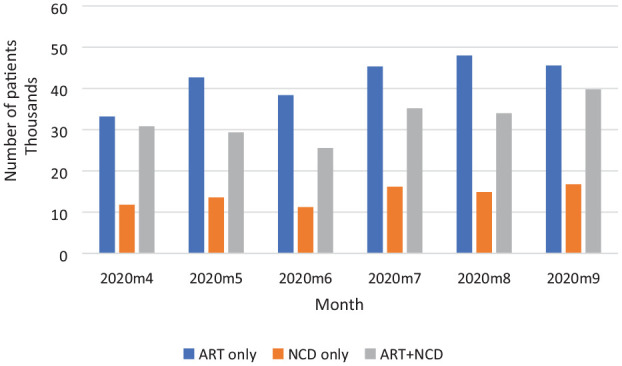
Number of Smart Locker PuP users, April to September 2020. Source: Authors’ compilation from Strydom.^36^

We find the decline in ART + NCD utilization surprising given that all patients in the 3 groups were allowed to use the “Smart Locker PuPs,” and the fact that the number of people receiving ART + NCD increased by 29% between April and September 2020 (Figure 3) under the “Smart Locker PuPs” in Gauteng and Free State Provinces.^36^ However, it may reflect the difficulty in managing multi-morbidities among patients with chronic diseases. Notably, previous studies have reported that the delivery of HIV and NCD services remains fragmented, with some patients attending HIV services at PHC clinics and NCD services at hospitals.^39^ Lack of coordination of care may result in multi-morbid patients experiencing challenges with their treatment schedules, which may feed into CCMDD service utilization. Thus, it is possible that the lockdown may have amplified the challenges experienced by patients with multi-morbidity and contributed to the decline in the medication pickups that we observed. The underlying mechanism for this effect, however, remains unclear. It is also worth noting that patients with multi-morbidities may have less resistance and higher vulnerability to acute health threats such as COVID-19.^40^ The burden of the pandemic in this group of patients may have led to a complex pattern in their use of health services such as those offered under the CCMDD program. Our findings are similar to previous studies, that the majority of patients utilising CCMDD were collecting ART-only medication, followed by NCD-only medication.^23^ This suggests that there is potential for further expansion of the utilisation of CCMDD by patients collecting ART + NCD medication, and the barriers to this expansion should be explored.

### Limitations of the study

There are limitations in this study. First, we were unable to obtain data on actual pickups or dispensed medicine parcels that were delivered hence the use of active patients on the CCMDD register to make inferences about utilization. The number of active patients may therefore not reflect actual utilization or pickups by patients. Secondly, we were unable to analyze the data at individual or facility-level given that the data is collected at the district level and aggregated for reporting by the National Department of Health. Another issue relates to the data quality. We observed some differences number of patients in January 2018^23^ and the dataset received for the current analysis which reflects challenges with data capturing within the CCMDD program. Many medication collection points do not have computers and/or internet connection to use the CCMDD software for program administration, which poses challenges in data capturing.^37^ Therefore, the results on ART + NCD may be due to challenges in data capturing associated with the program.

The population data used to obtain rates should be interpreted with caution since the actual district-level population estimates for 2018 to 2021 were unavailable. The population estimates used in the analysis relates to 2016 population data that were adjusted by the authors based on national population growth rates during the period under study. This may not reflect the true population estimates in the districts. Further, the dataset provides no information on patient characteristics such as age and sex which could help in explaining utilization patterns. Future studies should consider using such demographic variables, if available. Another limitation is that there was no power calculation of sample size in the study.

## Conclusion

This study examines the impact of COVID-19 lockdown on service utilization under the CCMDD program in South Africa. We found that service utilization among chronic patients was not impacted negatively. In particular, use of CCMDD services among patients receiving medications separately for either ART or NCDs increased during the lockdown restrictions implemented during April to September 2020. However, the number of patients simultaneously receiving medications for ART and chronic medications (referred to as ART + NCD) declined during the period. We also found that pre-lockdown trends in utilization are yet to be restored.

Although there was a positive effect of COVID-19 lockdown on ART-only and NCD-only service utilization for all socioeconomic groups, these effects were statistically insignificant. For ART + NCD, significant reductions occurred among poorer districts. In this context, patients should be educated about the need to continue with utilization of disease specific programs in the midst of a pandemic. Finally, the National Department of Health should consider equipping collection units with computers and internet to ensure that data is captured in real time to improve accuracy.

## Supplemental Material

sj-xlsx-1-his-10.1177_11786329231215040 – Supplemental material for The Impact of COVID-19 Lockdown on Service Utilization Among Chronic Disease Patients in South Africa Click here for additional data file. Supplemental material, sj-xlsx-1-his-10.1177_11786329231215040 for The Impact of COVID-19 Lockdown on Service Utilization Among Chronic Disease Patients in South Africa by Micheal Kofi Boachie, Mariana Khoza, Susan Goldstein, Maggie Munsamy, Karen Hofman and Evelyn Thsehla in Health Services Insights

sj-xlsx-2-his-10.1177_11786329231215040 – Supplemental material for The Impact of COVID-19 Lockdown on Service Utilization Among Chronic Disease Patients in South Africa Click here for additional data file. Supplemental material, sj-xlsx-2-his-10.1177_11786329231215040 for The Impact of COVID-19 Lockdown on Service Utilization Among Chronic Disease Patients in South Africa by Micheal Kofi Boachie, Mariana Khoza, Susan Goldstein, Maggie Munsamy, Karen Hofman and Evelyn Thsehla in Health Services Insights
